# Fructose-related metabolic stress in MASLD: integrating intestinal substrate partitioning, hepatic phosphorus metabolite dynamics, and the inflammatory-fibrotic interface

**DOI:** 10.3389/fmed.2026.1964793

**Published:** 2026-10-28

**Authors:** Lin Qi, Yang Du, Yunze Shi, Xingyuan Huang, Mingfeng Liu, Ying Fang, Xiaoyang Hu

**Affiliations:** 1 Graduate School, Heilongjiang University of Chinese Medicine, Harbin, Heilongjiang, China; 2 Basic Medical College of Heilongjiang University of Chinese Medicine, Harbin, Heilongjiang, China

**Keywords:** fibrotic niche, fructose, glycerol-3-phosphate, gut-liver axis, ketohexokinase, metabolic dysfunction-associated steatotic liver disease, phosphorus-31 magnetic resonance spectroscopy

## Abstract

Fructose intake is associated with metabolic dysfunction-associated steatotic liver disease (MASLD), yet similar levels of intake may produce divergent hepatic outcomes. Previous reviews have largely focused on ketohexokinase (KHK)-mediated fructose catabolism, *de novo* lipogenesis (DNL), uric acid metabolism, or the gut–liver axis, with less attention to the links among intestinal substrate partitioning, dynamic hepatic phosphorus metabolite responses, triacylglycerol (TAG) assembly, and disease stage. This review organizes these pathways within a dual-input, dual-timescale framework to distinguish dietary intake from effective hepatic exposure and acute metabolic responses from chronic disease outcomes, while identifying unvalidated links between these levels. Intact fructose and small-intestine-derived metabolites entering the portal circulation are classified as substrate-related inputs, whereas distal fructose spillover, microbial functional changes, barrier status, and microbe-derived molecules are classified as gut-derived signaling inputs. The framework distinguishes substrate supply relevant to hepatic KHK-fructose−1-phosphate (F1P) processing and TAG assembly from gut-derived signals potentially relevant to the inflammatory context; their independent causal contributions in human MASLD remain unestablished. These inputs may converge at the level of hepatocellular injury and the intrahepatic immune environment, but their covariance and temporal order have not been examined within a unified human study. Human fructose-challenge studies support KHK-dependent phosphomonoester and inorganic phosphate responses as pharmacodynamic readouts. This evidence does not establish comparable KHK dependence across the full adenosine triphosphate (ATP) time course or validate these acute responses as predictors of metabolic dysfunction-associated steatohepatitis (MASH) activity or fibrosis progression. The proposed roles of circulating glycerate, glycerol-3-phosphate (G3P)-free fatty acid (FFA) coupling, and microbial fructose buffering are supported primarily by experimental evidence. By separating fructose exposure, immediate metabolic responses, background amplifiers, and disease outcomes, this framework provides a testable basis for studies combining standardized fructose challenges, phosphorus-31 magnetic resonance spectroscopy (^31^P-MRS), stable-isotope tracing, and longitudinal disease outcomes.

## 1 Introduction

The term metabolic dysfunction-associated steatotic liver disease (MASLD) has shifted the clinical conceptualization of steatotic liver disease from an exclusion-based diagnosis toward a metabolically defined disease spectrum (1). Some patients progress to metabolic dysfunction-associated steatohepatitis (MASH), progressive fibrosis, cirrhosis, and related complications. Obesity, insulin resistance, dysregulated lipid metabolism, and adverse dietary exposures jointly influence disease evolution. Current guidelines continue to place weight management and improved diet quality at the center of care and specifically recommend reducing sugar-sweetened beverages and added sugars (2, 3).

Fructose is processed mainly in the small intestine and liver. In hepatocytes, fructose phosphorylation bypasses direct control by phosphofructokinase-1, the classic rate-limiting enzyme of glycolysis, and can therefore alter phosphorylated intermediates, inorganic phosphate, and adenosine triphosphate (ATP) turnover within minutes. Previous reviews have examined fructose metabolism, *de novo* lipogenesis (DNL), uric acid, and systemic metabolic effects (4–9). A recent KHK-focused review further synthesizes its molecular characteristics, regulatory networks, tissue-specific functions, and therapeutic potential (10). Together, these reviews provide the biochemical basis for examining why similar levels of fructose intake may produce different hepatic responses.

This review examines heterogeneity in fructose responses in MASLD through a dual-input, dual-timescale framework. Its contribution is to organize established pathways around the distinction between dietary intake and effective hepatic exposure, and between immediate metabolic responses and chronic disease outcomes. Substrate-related inputs and gut-derived signaling inputs are considered separately to distinguish their proposed contributions and identify where interactions remain untested. Within this framework, acute phosphorus metabolite responses and carbon flux are evaluated alongside the evidence for chronic hepatocellular injury and inflammatory–fibrotic remodeling. This organization clarifies which links are supported by human studies, which rely on experimental models, and which require validation before acute fructose responses can be interpreted in relation to MASH progression.

## 2 Intestinal substrate partitioning determines effective hepatic fructose exposure

### 2.1 The proximal small intestine as a determinant of fructose exposure

Hepatic fructose exposure does not directly mirror dietary intake. Before reaching the portal circulation, fructose is absorbed, phosphorylated, and oxidized by the small-intestinal epithelium and can be converted into glucose, lactate, glycerate, and other organic acids. At low-to-moderate loads, proximal intestinal processing reduces the proportion of intact fructose delivered to the liver or distal intestinal lumen. With high doses, rapid ingestion, or limited processing capacity, portal substrate delivery and distal spillover may increase simultaneously (11–13). Intestinal first-pass metabolism is therefore not a fixed protective mechanism; its effect depends on dose, ingestion rate, food matrix, and host metabolic capacity (14–17).

Tissue-specific KHK studies show that intestinal fructose handling has effects beyond substrate clearance. Jang et al. found that intestinal KHK-C deletion increased fructose delivery to the liver and microbiota and aggravated hepatic steatosis, whereas increased intestinal KHK-C expression reduced fructose spillover and lipogenesis (18). Andres-Hernando et al. distinguished an intestinal role in sugar intake from a hepatic role in sugar-induced metabolic dysfunction (19). More recent studies identified benefits of suppressing intestinal fructose metabolism: Lopez et al. reported reduced obesity and insulin resistance associated with decreased dietary fat absorption and ileal lacteal remodeling, while Song et al. reported reduced hepatic steatosis and improved glucose homeostasis after intestinal *Khk* deletion during high-fructose exposure (20, 21). These findings concern different exposure conditions, genetic manipulations, and endpoints. They indicate that reduced hepatic fructose delivery cannot be assumed to predict the overall metabolic effect of intestinal KHK activity. Intestinal fructose metabolism may therefore buffer hepatic exposure while also influencing sugar intake and dietary fat absorption; its net metabolic effect depends on the exposure conditions and the outcome examined.

This partitioning step generates two functional inputs. Substrate-related inputs include intact fructose entering the portal circulation and small-intestine-derived glucose and organic acids. Intact fructose directly determines the substrate load for hepatic KHK. Intestinal metabolites arise downstream of KHK and may supplement subsequent carbon flux, but their quantitative contribution to human glycerol-3-phosphate (G3P) supply and TAG assembly has not been established. Gut-derived signaling inputs originate from fructose that escapes proximal intestinal utilization and from its effects on microbial substrate use, barrier integrity, and the immune environment. The two inputs can coexist and need not change to the same extent.

### 2.2 Circulating glycerate as an experimental readout of intestinal fructose processing

Using genetically diverse mouse strains, metabolomics, and *in vivo* isotope tracing, Ramirez et al. found that the increase in circulating glycerate after oral fructose was associated with greater intestinal fructose catabolism and lower susceptibility to hepatic steatosis during chronic fructose exposure (22). These findings link proximal processing capacity to hepatic outcomes and suggest that a standardized challenge may provide more dynamic information than a static estimate of intake.

These findings were derived from a cross-strain mouse study. The reproducibility, reference range, dose–response relationship, and predictive performance of circulating glycerate have not been established in human MASLD. It is therefore considered an experimental readout for further investigation, rather than a marker ready for clinical stratification or treatment selection.

### 2.3 Exposure form, energy context, and the specificity of fructose effects

Isocaloric substitution and excess-energy intake address different questions. Isocaloric studies compare fructose with other carbohydrates under similar total energy intake, whereas hypercaloric studies alter both sugar load and energy balance. The latter more closely resemble excessive consumption of sugar-sweetened beverages, but any increase in liver fat is also influenced by the energy surplus. A systematic review of controlled feeding trials found no consistent effect on liver fat under isocaloric substitution, whereas high-dose fructose supplied as excess energy increased intrahepatic lipid and alanine aminotransferase (ALT) (23). These study designs should therefore be interpreted separately.

Pure fructose, sucrose, and high-fructose corn syrup differ in their glucose-to-fructose ratios and absorptive contexts. Co-ingested glucose can modify intestinal absorption, insulin responses, adipose-tissue lipolysis, and hepatic substrate partitioning. Pharmacodynamic findings from pure-fructose challenges therefore apply most directly to comparable exposure conditions (4, 6).

Liquid sugars are generally consumed more rapidly, resulting in faster gastric emptying and a more concentrated pulse of portal substrate delivery. In solid foods, mixed meals, and whole fruit, fiber, mastication, and the nutrient matrix alter absorption kinetics and satiety. A systematic review stratified by food source and energy control found that sugar-sweetened beverages, fruit juice, whole fruit, and other fructose-containing foods did not have uniform hepatic effects (24). Whole fruit therefore has a different exposure profile and physiological context from sugar-sweetened beverages or pure-fructose challenges.

A single 60- or 75-g fructose challenge is suitable for assessing dynamic phosphorus metabolite and pharmacodynamic responses (25, 26). Long-term habitual intake also involves energy balance, weight change, metabolic adaptation, microbiome remodeling, and disease stage. Acute challenges capture responses over minutes to hours, whereas habitual exposure reflects the integrated consequences of sustained intake. When discussing “fructose exposure,” this review therefore specifies, where possible, the dose, energy context, chemical form of the sugar, food matrix, ingestion rate, and duration.

Dietary intake is not the only source of fructose available for KHK-mediated metabolism. The polyol pathway converts glucose to sorbitol through aldose reductase and subsequently to fructose through sorbitol dehydrogenase. In mice, endogenous fructose production and its metabolism contributed to glucose-induced fatty liver and metabolic dysfunction, providing experimental evidence for a substrate source beyond dietary fructose (27).

Andres-Hernando et al. further showed that ethanol activated endogenous fructose production through the polyol pathway and that KHK-dependent metabolism contributed to alcohol-associated liver disease in mice (28). Although this alcohol-related evidence cannot be directly extrapolated to MASLD, it broadens the substrate context of the present framework: hepatic KHK may process both dietary and endogenously produced fructose. The quantitative contribution of endogenous fructose to human MASLD, independently of dietary fructose intake, remains unresolved.

## 3 KHK-related hepatic phosphorus metabolite dynamics: acute processing and pharmacodynamic readouts

### 3.1 A standardized fructose challenge provides a time-resolved window into hepatic responses

Phosphorus-31 magnetic resonance spectroscopy (^31^P-MRS) can serially monitor hepatic phosphomonoester (PME), inorganic phosphate (Pi), and ATP-related signals before and after a fructose challenge. In this review, “dynamic hepatic phosphorus metabolite responses” refers to the amplitude, time to peak, recovery pattern, and coordination of these signals. The term does not refer to static phosphorus content or irreversible energy depletion.

Bril et al. enrolled 37 participants with overweight or obesity but without diabetes and monitored them for 60 min after a 75-g oral fructose challenge. Baseline ATP, PME, and phosphodiester-related signals did not differ significantly between groups. Participants without MASLD showed a rapid increase in PME, an early fall and subsequent recovery in Pi, and a decline in ATP. In participants with MASLD, the PME and Pi responses were attenuated, and the ATP time course also differed from that observed in the non-MASLD group (25). These findings provide an exploratory observation of differences in hepatic phosphorus metabolite responses between participants with and without MASLD. The small, cross-sectional study cannot establish a characteristic MASLD response profile, its underlying mechanism, or its relationship to disease progression. The observed differences could reflect substrate delivery, fructose phosphorylation, metabolite turnover, or energy regeneration. Without independent replication, histological endpoints, and longitudinal outcomes, this study informs hypotheses about response heterogeneity rather than validating the proposed framework.

### 3.2 The biological significance of an attenuated response in MASLD remains uncertain

The attenuated response to fructose loading in MASLD may arise at several levels. Portal fructose delivery, hepatocellular KHK flux, turnover of F1P and other phosphomonoesters, Pi reutilization, and ATP regeneration can each alter the response curve. Metabolic adaptation to long-term nutrient exposure may also reshape the short-term response. Another possibility is reduced metabolic flexibility, in which the liver cannot mount the normal amplitude or temporal pattern when substrate delivery rises abruptly. Cross-sectional data cannot distinguish among these mechanisms. It therefore remains unclear whether attenuation reflects adaptive protection, limited processing capacity, or disease-related metabolic dysfunction.

Analyses of the complete time course may be more informative than a single peak value. Areas under the PME and Pi curves, time to peak, ATP recovery rate, and coupling among signals may better represent immediate processing capacity. Reproducibility across repeated challenges also requires standardization of the preceding diet and fasting duration, together with measurement or control of hepatic glycogen and baseline liver fat. The direction of risk should be determined from the longitudinal relationships of response amplitude, timing, and recovery with liver fat or histological outcomes rather than assumed from response strength alone.

### 3.3 KHK inhibition establishes the pharmacodynamic dependence of PME and pi responses, whereas the ATP response and its prognostic significance remain unresolved

The highly active C isoform of ketohexokinase (KHK-C) rapidly phosphorylates fructose to F1P. When the initial phosphorylation rate transiently exceeds F1P cleavage and ATP regeneration, phosphate can enter F1P and other PME pools, with accompanying changes in Pi and ATP turnover (4, 5, 29). This mechanism accounts for the minute-scale phosphorus metabolite signals observed after a fructose challenge. The metabolic effects of KHK also depend on its isoform. KHK-C is expressed predominantly in the liver, intestine, and kidneys and has a high affinity for fructose, whereas KHK-A is more widely distributed and has a lower affinity. Ishimoto et al. found that deletion of both isoforms prevented fructose-induced metabolic syndrome in mice, while deletion of KHK-A alone exacerbated it. The authors attributed the protective effect of KHK-A to reduced fructose availability for hepatic KHK-C-mediated metabolism (30). These findings distinguish the effects of the two isoforms and support considering isoform selectivity when interpreting KHK-targeted interventions. Whether the same balance operates in human MASLD remains uncertain.

Tissue-specific deletion studies provide complementary evidence for the role of hepatic KHK in chronic metabolic dysfunction. Andres-Hernando et al. showed that liver-specific KHK deletion protected mice against sugar-induced metabolic syndrome, distinguishing hepatic metabolic effects from the intestinal regulation of sugar intake (19). In mice exposed to a 20% fructose drinking solution, Yang et al. found that liver-specific *Khk* deletion reduced hepatic steatosis, liver injury, and disturbances in lipid and uric acid metabolism. It also reduced fructose-stimulated expression of KHK, fructose-related transporters, and aldolase B in the intestine and kidneys (31). These findings support a hepatic contribution to systemic fructose-related dysfunction and indicate coordinated changes in extrahepatic fructose handling. They do not identify a specific liver-to-intestine-to-kidney signaling mediator or establish equivalent effects in human MASLD.

Koene et al. primarily evaluated the safety and efficacy of the KHK inhibitor PF-06835919 in hereditary fructose intolerance. The investigators first performed hepatic pharmacodynamic validation in 14 participants with overweight or obesity and MASLD. PF-06835919 suppressed the PME peak and transient Pi decline after a 60-g oral fructose challenge, directly supporting the dependence of both signals on KHK-mediated fructose phosphorylation (26). The study did not provide comparable pharmacodynamic evidence across the full ATP time course and was not designed as a therapeutic trial of histological improvement in MASLD. The Bril and Koene studies address different questions: the former describes between-group response differences, whereas the latter tests the effect of KHK inhibition on acute PME and Pi responses. The pharmacodynamic findings do not independently replicate the MASLD-associated response pattern reported by Bril et al. Together, these studies support investigation of dynamic hepatic responses, but they do not establish a validated response phenotype or a link to MASH progression.

Longer-term clinical studies suggest that KHK inhibition can alter liver fat and selected metabolic or inflammatory measures, although acute ^31^P-MRS was not a primary endpoint (32, 33). PME and Pi responses can currently be used as hepatic pharmacodynamic readouts of KHK inhibition, whereas ATP changes reflect the broader energetic response to fructose processing. Their relationships with histological improvement in MASH, fibrosis regression, or long-term clinical outcomes remain unknown.

### 3.4 Technical limitations in the interpretation of ^31^P-MRS signals and challenge protocols

Although ^31^P-MRS can noninvasively capture temporal changes in hepatic phosphorus metabolites after a fructose challenge, individual spectral peaks do not correspond to single metabolites. PME contains several phosphomonoesters. Its increase after fructose loading is mechanistically consistent with F1P formation, but it remains a composite signal and cannot provide metabolite-specific quantification of F1P. ATP and Pi measurements are also affected by peak fitting, signal-to-noise ratio, the choice of reference, field strength, coil configuration, and acquisition sequence. Standardized calibration and acquisition are required when absolute concentrations are compared across studies (34).

Respiratory liver motion, breath-hold compliance, voxel placement, regional steatosis, and tissue heterogeneity can affect reproducibility. A methodological study at 3 T reported acceptable within-day and between-day reproducibility for absolute hepatic *γ*-ATP and Pi quantification, although coil configuration, participant repositioning, and the quantification strategy remained influential (34). Greater temporal resolution often comes at the expense of spatial resolution or signal-to-noise ratio and may therefore underestimate brief peaks. The pre-challenge diet, fasting duration, hepatic glycogen stores, physical activity, and baseline liver fat can also alter the response curve. Future studies should report voxel placement, peak fitting, internal or external references, sampling intervals, reproducibility metrics, and the handling of missing data.

Current single-dose oral fructose protocols using 60 or 75 g are intended mainly to generate clear dynamic and pharmacodynamic signals, and their generalizability is largely confined to similar experimental conditions. Typical mixed meals, solid foods, and habitual intake produce different patterns of substrate delivery. Future studies should compare doses, ingestion rates, and liquid versus solid forms while standardizing fasting and sampling protocols. On current evidence, ^31^P-MRS is most appropriately used for mechanistic studies and pharmacodynamic validation; its role in clinical risk stratification requires independent validation.

## 4 From fructose carbon flux to TAG assembly: DNL, the glycerol backbone, and peripheral FFA flux

### 4.1 DNL primarily accounts for newly synthesized fatty acids

Aldolase B cleaves F1P into dihydroxyacetone phosphate (DHAP) and glyceraldehyde. These three-carbon intermediates can enter the pyruvate–citrate–cytosolic acetyl-CoA pathway and support a lipogenic enzyme network that includes ATP citrate lyase, acetyl-CoA carboxylase, fatty acid synthase, and stearoyl-CoA desaturase 1 (35). A randomized controlled study in humans showed that sustained consumption of fructose- or sucrose-sweetened beverages increased hepatic DNL without a marked increase in total energy intake, whereas glucose-sweetened beverages did not show the same effect (36).

DNL explains how new fatty acids are synthesized, whereas the retention of pre-existing fatty acids in the liver is a separate process (37). A classic human tracer study showed that, in patients classified under the former non-alcoholic fatty liver disease (NAFLD) nomenclature, a substantial proportion of the fatty acids in hepatic TAG originated from circulating non-esterified fatty acids, with DNL providing another important source (38). Recent reviews likewise emphasize that hepatic lipid accumulation in MASLD reflects the combined effects of circulating fatty acid input, DNL, fatty acid oxidation, and lipoprotein export (39). Adipose-tissue insulin resistance weakens the suppression of lipolysis, making persistently increased circulating free fatty acid (FFA) flux an important substrate source for hepatic re-esterification (40).

In aldolase B (ALDOB) deficiency, suppressing hepatic DNL-related pathways reduced *de novo* fatty acid synthesis without a parallel reduction in intrahepatic TAG accumulation. This finding further indicates that changes in DNL do not map directly onto net hepatic lipid storage (41).

Hepatic TAG accumulation also depends on lipid disposal. Fatty acids can undergo oxidation, while TAG can be packaged into very-low-density lipoprotein (VLDL) particles and exported. Increased TAG synthesis therefore need not result in a proportional increase in hepatic storage if disposal also increases; conversely, insufficient export can favor lipid retention. VLDL secretion is influenced by substrate availability, insulin action, and lipoprotein assembly, and these processes vary across metabolic and genetic contexts (39). Plasma TAG concentrations cannot resolve these pathways because they reflect both lipoprotein secretion and peripheral clearance. For fructose-response studies, changes in DNL, hepatic fat, and circulating TAG should therefore be interpreted alongside measures of fatty acid oxidation and VLDL-TAG kinetics, rather than used interchangeably as indicators of hepatic lipid accumulation.

### 4.2 Experimental evidence for context-dependent G3P-FFA coupling

G3P is a major source of the glycerol backbone of TAG, and DHAP can be reduced to G3P. Fructose carbon does not need to enter fatty acid chains in large amounts to promote TAG formation. By increasing glycerol-backbone availability, fructose-derived carbon may facilitate the esterification of pre-existing FFAs into TAG. The proposed coupling concerns whether fructose-derived glycerol-backbone supply facilitates TAG assembly when peripheral FFA availability is high. Its quantitative importance in common human MASLD remains to be established.

Tao et al. studied mice with liver-specific double knockout of Irs1 and Irs2, a model of complete hepatic insulin resistance, under either a Gubra–Amylin NASH (GAN) diet, whose name retains the former term non-alcoholic steatohepatitis (NASH), or a diet containing 60% fructose. The model was accompanied by follistatin (FST)-associated adipose-tissue insulin resistance and increased lipolysis, which raised circulating FFA availability. Inhibiting adipose-tissue lipolysis markedly reduced acute hepatic lipid formation. Stable-isotope tracing showed that fructose-derived carbon contributed more to the glycerol backbone of hepatic TAG than to its fatty acid chains, suggesting that high FFA flux and glycerol-backbone supply can become coupled in a specific metabolic context (42).

Acute hepatic lipid formation persisted after hepatocyte KHK-C deletion, suggesting compensation by extrahepatic fructose metabolites or other sources of G3P. The specific sources remain unclear because the available carbon-flux tracing did not resolve all bypass pathways under this condition. Complete hepatic insulin resistance, FST-associated lipolysis, the multicomponent GAN diet, and the high proportion of dietary fructose jointly defined the experimental context and limited the applicability of these findings to common human MASLD. Vacca et al.'s comparative ranking of murine dietary models against human MASLD further supports evaluating model relevance beyond the presence of steatosis alone (43). For the present framework, model selection should match the metabolic or disease-stage feature being tested; reproducing lipid accumulation does not by itself validate the model for studying human inflammatory or fibrotic progression. Within this model, the findings support a contribution of fructose-derived glycerol-backbone supply to TAG assembly under conditions of high FFA availability. They do not establish the magnitude or prevalence of this contribution in common human MASLD. G3P–FFA coupling is therefore included in the framework as a context-dependent hypothesis for testing, rather than an established dominant route of fructose-related hepatic lipid accumulation. Human tracer studies should determine its contribution relative to DNL and other lipid fluxes.

### 4.3 Transcriptional programs extend substrate effects

DNL, G3P, and FFA re-esterification primarily describe substrate flux. Carbohydrate-responsive element-binding protein (ChREBP) responds to sugar-derived metabolic intermediates and regulates multiple lipogenic genes (44, 45). Sterol regulatory element-binding protein-1c (SREBP-1c) contributes to insulin- and nutrient-dependent control of lipid synthesis, but it is not treated here as a fructose-specific transcriptional readout (4). Hepatic ChREBP deficiency alters fructose-related metabolic phenotypes in pregnant mice and their female offspring, although this specific physiological setting limits extrapolation to common MASLD (46).

In the context of citrin deficiency, Tiwari et al. showed that *SLC25A13 loss* caused G3P accumulation and activation of ChREBP-dependent fibroblast growth factor 21 (FGF21) transcription and lipogenic programs (47). This study provides mechanistic evidence linking G3P to transcriptional regulation in a specific genetic and metabolic setting. It does not directly establish that dietary fructose activates the same pathway in common MASLD; its relevance here is to identify a possible connection between glycerol-backbone metabolism and metabolic sensing for further testing.

## 5 Context-dependent translation of metabolic stress into hepatocellular injury

### 5.1 Nucleotide degradation and uric acid as candidate amplification pathways

When KHK-mediated ATP turnover increases, adenosine monophosphate (AMP) may be rephosphorylated or enter salvage pathways; alternatively, it can undergo AMP deamination and purine degradation to generate hypoxanthine, xanthine, and uric acid. Uric acid production is one branch of phosphate-related metabolic stress and is not the only route through which changes in ATP turnover may contribute to liver injury (4, 48).

The effects of uric acid depend on its concentration, subcellular localization, and inflammatory context. Extracellular uric acid has antioxidant properties, whereas high concentrations or intracellular accumulation may participate in oxidative and inflammatory signaling. Serum uric acid does not necessarily reflect intracellular uric acid exposure in hepatocytes. Cohort analyses have reported associations between serum uric acid and NAFLD but have not established a clear causal relationship (49). Its role in MASLD is further influenced by intracellular versus extracellular distribution and the underlying metabolic and inflammatory state (50). In animal models, inhibiting uric acid production attenuated some forms of high-fructose-related liver injury (51). Current evidence therefore supports uric acid as a context-dependent modifier of fructose-related injury rather than an obligatory mediator of MASH development.

### 5.2 Suppressed fatty acid oxidation and organelle stress as parallel stressors

Reactive oxygen species (ROS) production can arise from xanthine oxidoreductase activity, nicotinamide adenine dinucleotide phosphate (NADPH) oxidase activity, and stress within the mitochondrial electron transport chain. In specific animal models, including copper deficiency and diabetes, high-fructose exposure aggravates oxidative stress, lipid peroxidation, and endoplasmic reticulum (ER) stress (51, 52). These processes can occur in parallel and do not necessarily depend on uric acid. High-fructose exposure may also suppress fatty acid oxidation and thereby reduce the hepatic capacity to dispose of excess lipid substrate (53).

Using cultured hepatocytes and mouse models, Helsley et al. found that KHK-C remodeled global protein acetylation, reduced the stability of carnitine palmitoyltransferase 1A (CPT1A), and thereby impaired CPT1A-mediated fatty acid oxidation (54). These experiments identify a proteostatic link between KHK-C and fatty acid oxidation, but do not establish its quantitative contribution to lipid accumulation or disease progression in human MASLD.

In diet-induced and genetic NAFLD models, Park et al. found that fructose-induced KHK-C expression enhanced endoplasmic-reticulum stress and promoted liver injury partly independently of its lipogenic effects (55). Because the evidence comes from experimental models, its relationship to disease stage in human MASH remains uncertain.

The relationship between KHK and ER stress is not uniform across experimental settings. Park et al. linked increased KHK-C expression to ER stress and liver injury in diet-induced and genetic models (55), whereas Elsaid et al. identified a role for KHK in maintaining adaptive IRE1*α*-XBP1 signaling under nutrient stress. In the latter study, KHK knockdown increased protein aggregation and shifted signaling toward proapoptotic PERK–CHOP responses (56). These findings raise the possibility that KHK can contribute to metabolic stress while also supporting the cellular response to that stress. They do not establish why the experimental outcomes differ: nutrient composition, exposure duration, model characteristics, and the extent of KHK reduction should be compared directly. Human liver expression data provide observational support for the association between KHK and unfolded protein response pathways, but do not establish the consequences of therapeutic KHK reduction in patients (56).

Hormonal regulation may also modify hepatic fructose handling. In a preprint, Elsaid et al. reported reduced hepatocyte KHK expression in diet-induced and genetic models of MASLD and identified growth hormone (GH)/insulin-like growth factor-1 (IGF-1) signaling as a potential regulator of KHK abundance. Enhanced GH signaling was associated with lower KHK expression in mice, while GH and IGF-1 treatment promoted KHK degradation in liver cell cultures (57). These findings suggest a potential adaptive mechanism that limits hepatic fructose metabolism and may contribute to differences in hepatic responses at comparable intake levels. Whether this response provides sustained protection against human MASLD remains unresolved.

Park et al. compared liver-targeted KHK siRNA with systemic PF-06835919 in male mice. Both reduced steatosis, but knockdown suppressed DNL-related programs, whereas the inhibitor enhanced fatty acid oxidation. PF-06835919 also affected triokinase/FMN cyclase (TKFC), with F1P and glycogen accumulation, hepatomegaly, and no improvement in glucose tolerance (58). The comparison distinguishes reducing KHK protein from inhibiting its catalytic activity, although tissue targeting and compound selectivity also differed. The adverse findings should therefore not be generalized to all KHK inhibitors. Compensatory increases in mitochondrial respiration after an acute challenge should also be distinguished from structural and functional injury during chronic exposure.

### 5.3 The inflammasome as a convergence point for multiple signals

When hepatocytes can no longer buffer metabolic and organelle stress, they may release sterile inflammatory signals, including extracellular RNA, that promote cytokine production and activate Kupffer cells and monocyte-derived macrophages (59). Other danger-associated molecules, extracellular vesicles, and lipid peroxidation products may also participate, although their evidence base and disease-stage relevance need to be considered separately.

A study in rats fed a high-fructose diet suggested reciprocal amplification among NOD-like receptor family pyrin domain-containing 3 (NLRP3) activation, oxidative stress, and endoplasmic-reticulum stress (60). In the broader inflammasome pathway, NLRP3 activation promotes caspase-1 activation, maturation of interleukin-1β and interleukin-18, and gasdermin D-mediated pyroptotic signaling (61). In MASLD, NLRP3 may also connect microbiota-derived signals with metabolic inflammation (62). Within the present framework, NLRP3 serves as a point of convergence for metabolic and immune signals rather than a fructose-specific pathway.

Whether early phosphorus metabolite changes translate into hepatocellular injury is not determined by PME or ATP alone. Nucleotide degradation, fatty acid supply, redox status, organelle compensation, and gut-derived stimuli jointly determine the threshold for injury.

## 6 Gut-derived signals as a parallel modifying input

### 6.1 Distal fructose spillover and functional adaptation of the microbiota

Fructose that escapes proximal intestinal utilization can reach more distal intestinal segments and alter available substrates and microbial metabolites. Microbial handling of fructose is not uniform. Some microbial functional profiles may enhance substrate clearance, whereas other ecological changes may accompany barrier instability. The broad claim that “high fructose inevitably causes dysbiosis” obscures differences in dietary context, microbial function, and host state (63–66). Human intervention findings qualify the effects reported in experimental models. In a controlled crossover pilot study, Alemán et al. found no detectable changes in fecal microbiota, intestinal permeability, or markers of endotoxemia after 14 days of fructose exposure in participants with obesity (64). The controlled dietary setting strengthens interpretation of this short-term result, while the small sample and brief exposure limit conclusions about sustained intake or disease progression. Differences in species, background diet, exposure duration, and measured endpoints may contribute to the discrepancy with rodent studies, but have not been established as its explanation. The available evidence therefore does not support a universal sequence from fructose intake to dysbiosis and barrier failure; microbial and barrier responses should be measured rather than assumed.

In male mice, Jung et al. found that an intestinal microbial community adapted to dietary fiber enhanced dietary fructose clearance, improved insulin resistance and hepatic steatosis, and reduced the expression of selected hepatic fibrosis-related genes (67). The findings arose from a specific high-fructose and dietary-fiber intervention model. Their applicability to human MASH and to clinically tolerable doses of dietary fiber remains uncertain.

Host intestinal fructose metabolism can also influence microbial effects on nutrient absorption. In the Lopez study, suppression of intestinal fructose catabolism altered the microbiome, reduced ileal macrophages supporting lacteal growth, and decreased dietary fat absorption (20). Song et al. also reported improved colonic epithelial barrier integrity after intestinal *Khk* deletion (21). Thus, gut-derived effects may involve changes in nutrient absorption as well as inflammatory signaling; neither can be inferred from fructose spillover alone.

### 6.2 Barrier integrity shapes the portal inflammatory context

Previous reviews have summarized the associations of intestinal barrier injury and microbiome alterations with steatotic liver disease (68–71). Animal studies further indicate that high-fructose and high-fat diets can induce chronic intestinal inflammation through microbiome alterations (72, 73). Loss of barrier proteins and translocation of pathogen-associated molecular patterns can increase inflammatory input through the portal circulation. Lipopolysaccharide, lipoteichoic acid, and other microbial molecules may thereby provide persistent, low-grade immune stimulation.

Substrate-related inputs primarily affect KHK load and phosphorus metabolite turnover, whereas barrier instability changes the immune context in which the liver encounters the same metabolic stress. The two processes need not change in parallel; their independent contributions in human MASLD remain unestablished. A stronger inflammatory response does not necessarily coincide with a larger acute decline in ATP.

### 6.3 Human monocyte sensitization provides a metabolic–immune interface

In a human study, Staltner et al. found that fructose intake enhanced the pro-inflammatory response of monocytes from healthy participants to lipoteichoic acid and increased Toll-like receptor 2 mRNA expression. KHK inhibition attenuated this response (74). The findings indicate that fructose can alter the response threshold of peripheral innate immune cells to a microbial signal.

The participants were healthy, and the study assessed responses in peripheral monocytes, which are distinct from the intrahepatic macrophage lineages present in MASH. The study provides a human mechanistic interface between a metabolic substrate and a microbial stimulus, but its conclusions are limited to the responsiveness of peripheral innate immune cells.

### 6.4 Cross-organ coordination of fructose handling

The intestine, liver, and kidneys should also be considered as interacting sites of fructose handling. Liver-specific *Khk* deletion altered intestinal and renal fructose-handling pathways in the Yang study, whereas intestinal *Khk* deletion improved hepatic and systemic metabolic outcomes in the Song study (21, 31). Yi et al. additionally reported that Pax8-rtTA/LC1-driven inducible *Khk* deletion improved hepatic steatosis, glucose homeostasis, and colonic barrier integrity during high-fructose exposure (75). These mouse studies support evaluating fructose metabolism across organs rather than treating intestinal and hepatic responses in isolation. However, coordinated changes across tissues do not by themselves identify the mediators or direction of interorgan communication.

Within the framework, cross-organ coordination may modify substrate exposure and the inflammatory context. It should be distinguished from direct evidence for gut-derived immune signaling, and its contribution to human MASLD remains to be established.

## 7 The inflammatory-fibrotic niche as a downstream context for fructose-related stress

The inflammatory–fibrotic niche of MASH connects hepatocellular injury with immune responses and tissue remodeling. Injured hepatocytes release danger signals that can alter Kupffer-cell and recruited monocyte-derived macrophage responses. Immune-cell mediators, including transforming growth factor-*β* (TGF-*β*), can promote hepatic stellate cell (HSC) activation and extracellular matrix production. These interactions are not uniformly fibrogenic: macrophage functions also include clearance of injured cells and support for tissue repair, and their effects depend on cell state and disease stage (76, 77). Fibrosis therefore reflects the balance between persistent injury, repair, matrix deposition, and matrix removal, rather than inflammatory activation alone.

Within the proposed framework, fructose-related metabolic stress may contribute to hepatocyte injury, while gut-derived signals may modify immune responsiveness. These inputs could influence the cellular interactions described above, but evidence of altered lipid metabolism or peripheral inflammation cannot by itself establish intrahepatic fibrogenesis. Demonstrating this connection requires evidence that changes in fructose exposure or handling alter hepatic immune–HSC interactions and matrix remodeling under defined conditions. The general mechanisms of MASH fibrosis provide a biological context for this hypothesis, rather than proof of a fructose-specific fibrotic pathway.

Human spatial multi-omics has identified the proximity of macrophages, activated HSCs, and endothelial cells in fibrotic regions and proposed candidate cell–cell interactions (78). These observations describe the tissue architecture that a fructose-related mechanism would need to explain, but they do not connect fructose exposure to its formation or maintenance.

Single-cell studies in mouse MASH and multimodal analyses of human HSCs provide complementary information on stromal cell states and candidate fibrogenic targets (79, 80). Neither establishes fructose-specific regulation of these programs. Their role in this review is to identify downstream cellular features against which proposed fructose-related effects could be tested, rather than to validate the upstream links.

The unresolved question is whether defined fructose exposure or changes in its handling alter these cell states and interactions, and whether such effects depend on disease stage. Representative studies and their evidence limitations are summarized in Table 1.

**Table 1:** Representative evidence for fructose-related metabolic and inflammatory pathways in MASLD.

Study (year) and DOI	Population or model and exposure/intervention	Methods	Main findings	Mechanistic relevance	Evidence type and limitations
Bril et al. (2026); 10.1210/clinem/dgag125	37 adults with overweight or obesity and without diabetes; MASLD and non-MASLD groups; 75-g oral fructose challenge with 60-min follow-up	Hepatic ^31^P-MRS with serial asssessment of PME, Pi, ATP, and phosphodiester-related signals	MASLD was associated with a distinct post-fructose time course. PME and Pi responses were attenuated, and the ATP time course differed from that in participants without MASLD.	Shows that acute hepatic phosphorus-metabolite responses to fructose differ by MASLD status.	Human observational challenge study. Cross-sectional design and limited sample size; no histological MASH activity, fibrosis progression, or longitudinal outcome; the biological meaning of attenuation remains uncertain.
Koene et al. (2025); 10.1172/JCI187376	Pharmacodynamic validation in 14 adults with overweight or obesity and MASLD; PF-06835919 versus placebo before a 60-g oral fructose challenge	Hepatic ^31^P-MRS before and after pharmacological KHK inhibition	KHK inhibition suppressed the PME peak and transient Pi decline after fructose loading.	Links PME and Pi responses to KHK-mediated fructose phosphorylation in humans.	Human pharmacodynamic intervention study. The main trial context was hereditary fructose intolerance; comparable pharmacodynamic evidence was not established for the complete ATP time course; not a MASLD histology trial.
Koene et al. (2026); 10.1038/s42255-026-01626-5	15 participants with MASLD; six-week PF-06835919 treatment versus placebo in a randomized crossover trial	Assessment of hepatic, peripheral, and adipose tissue insulin sensitivity, intrahepatic lipid, and substrate oxidation	Treatment improved hepatic insulin sensitivity and reduced intrahepatic lipid without changing body weight; peripheral and adipose tissue insulin sensitivity and nocturnal fat oxidation also improved.	Extends human KHK-inhibition evidence beyond acute phosphorus-metabolite responses to hepatic and systemic metabolic outcomes.	Human randomized placebo-controlled crossover trial. Small sample and short treatment duration; the reported metabolic benefits do not establish histological MASH improvement or fibrosis regression.
Ramirez et al. (2025); 10.1016/j.cmet.2025.03.017	Genetically diverse mouse strains exposed to oral fructose challenge and chronic fructose feeding	Metabolomics, *in vivo* isotope tracing, and cross-strain comparison	A stronger circulating glycerate response was associated with greater intestinal fructose catabolism and lower susceptibility to chronic fructose-induced hepatic steatosis.	Suggests that proximal intestinal fructose catabolism may influence effective hepatic substrate exposure.	Mouse experimental study. Animal evidence; reproducibility, reference range, dose-response behavior, and outcome prediction in human MASLD remain to be established.
Tao et al. (2025); 10.1038/s41467-025-66296-5	Liver-specific Irs1/Irs2 double-knockout mice under GAN diet or 60% fructose diet; lipolysis intervention and hepatocyte KHK-C deletion	Stable-isotope tracing of TAG glycerol-backbone and fatty-acid-chain carbon flow	Fructose-derived carbon preferentially supported the TAG glycerol backbone; high FFA flux coupled with glycerol-backbone supply in a specific insulin-resistant context.	Broadens TAG assembly beyond DNL by connecting fructose-derived glycerol-backbone supply with peripheral FFA availability.	Mouse mechanistic study. Extreme mouse model with complete hepatic insulin resistance, FST-associated lipolysis, and very high fructose exposure; human quantitative validation is lacking.
Jung et al. (2025); 10.1038/s42255-025-01356-0	Male mice with high-fructose exposure and dietary-fiber/microbiome adaptation interventions	Microbiota transfer, metabolic tracing, and hepatic phenotype assessment	A fiber-adapted microbiome increased intestinal fructose clearance, improved insulin resistance and steatosis, and reduced selected hepatic fibrotic marker genes.	Indicates that microbial fructose buffering can alter distal spillover and downstream metabolic phenotypes in experimental models.	Mouse intervention and mechanistic study. Specific mouse and dietary-fiber model; progressive histological fibrosis was not established; human MASH relevance and clinically tolerable intervention doses require validation.
Staltner et al. (2025); 10.1016/j.redox.2025.103729	Healthy human participants and peripheral monocytes; fructose intake, lipoteichoic-acid stimulation, and KHK inhibition	Peripheral monocyte inflammatory-response assays, TLR2-related measures, and pharmacological intervention	Fructose enhanced the monocyte response to lipoteichoic acid, and KHK inhibition attenuated this response.	Provides human evidence that fructose exposure may modify responsiveness to microbe-derived immune stimuli.	Human exposure study and peripheral-cell experiments. Participants were not patients with MASH; peripheral monocytes cannot be assumed to represent intrahepatic Kupffer cells or monocyte-derived macrophages.
Li et al. (2025); 10.1038/s41588-025-02407-8	Human liver tissue from healthy, MASLD, and fibrotic MASH states; no direct fructose exposure experiment	Spatial transcriptomics, single-cell profiling, spatial metabolomics, and cell-neighborhood analysis	Fibrotic regions contained coordinated macrophage, activated-HSC, and endothelial-cell programs, with candidate communication pathways such as RSPO3-LGR6.	Defines human spatial-cellular features of the inflammatory-fibrotic niche in MASH.	Human tissue observational study. Observational and computationally inferred; does not establish fructose specificity or causal regulation by fructose exposure.
Bendixen et al. (2024); 10.1016/j.jhep.2023.11.001	Advanced murine MASH model; no direct fructose exposure experiment	Single-cell transcriptomics of stromal-cell states and candidate signaling modules	Identified state changes in HSCs, pericyte-like cells, and other mesenchymal populations, including homeostatic or repair-related signaling modules.	Provides experimental context for downstream stromal-cell states involved in inflammatory-fibrotic remodeling.	Mouse single-cell profiling study. Species, diet model, and disease trajectory differ from human MASH; the study does not test fructose-related inputs directly.
Kim et al. (2025); 10.1016/j.jhep.2024.10.044	Human HSCs and human liver tissue, with liver-spheroid or mouse functional validation; no direct fructose exposure experiment	Single-nucleus transcriptomics, chromatin accessibility, multimodal analysis, and genetic/pharmacological validation	Identified MASH-enriched activated-HSC populations and candidate targets including PAI-1 (SERPINE1).	Links human fibrogenic cell states with selected functionally tested candidate molecules.	Human tissue profiling with experimental validation. Human tissue observation and model-based interventions must be interpreted separately; it does not prove fructose-specific regulation of HSC programs.

31 P-MRS, phosphorus-31 magnetic resonance spectroscopy; PME, phosphomonoester; Pi, inorganic phosphate; ATP, adenosine triphosphate; KHK, ketohexokinase; G3P, glycerol-3-phosphate; FFA, free fatty acid; TAG, triacylglycerol; HSC, hepatic stellate cell; GAN, Gubra-Amylin NASH; MASLD, metabolic dysfunction-associated steatotic liver disease; MASH, metabolic dysfunction-associated steatohepatitis; DNL, *de novo* lipogenesis; KHK-C, ketohexokinase isoform C; IRS1/IRS2, insulin receptor substrate 1/2; FST, follistatin; TLR2, Toll-like receptor 2; RSPO3, R-spondin 3; LGR6, leucine-rich repeat-containing G protein-coupled receptor 6; PAI-1, plasminogen activator inhibitor 1; SERPINE1, serpin family E member 1.

## 8 A dual-input, dual-timescale mechanistic framework

Based on the evidence described above, this review proposes a dual-input, dual-timescale working framework that requires validation. The framework is intended to explain why similar fructose intake may produce different hepatic responses; it does not imply that a complete human causal chain from fructose intake to MASH fibrosis has been established. The overall architecture of this framework, including the separation of substrate-related and gut-derived signaling inputs across acute and chronic timescales, is summarized in Figure 1.

**Figure 1:**
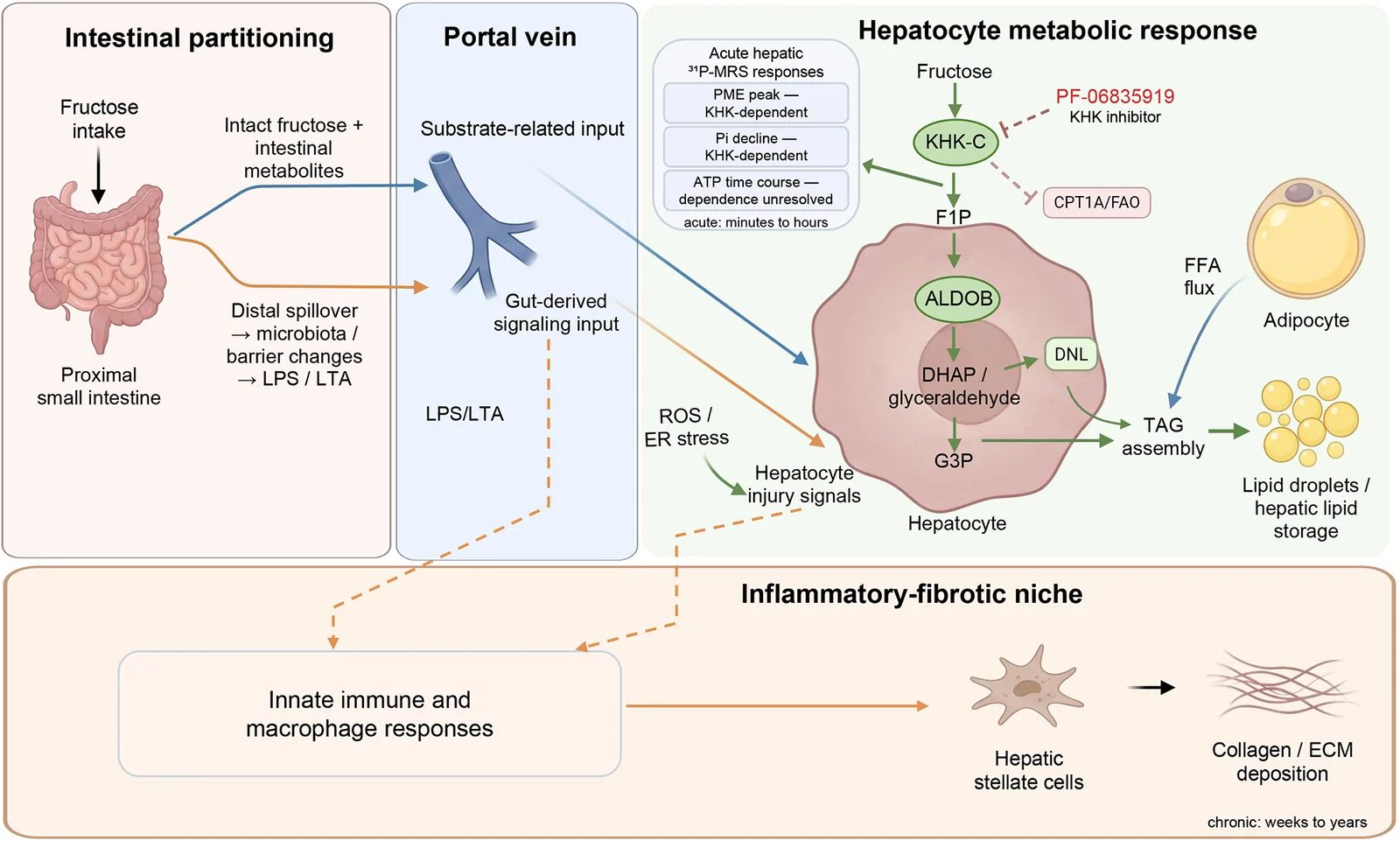
Dual-yinput and dual-timescale framework of fructose-related metabolic stress in MASLD. Proximal intestinal processing influences intact fructose and intestinal metabolite delivery to the liver, while distal spillover may affect microbial function and barrier-related signaling. These categories organize proposed substrate and signaling roles; they do not imply independently established causal effects in human MASLD. The hepatic module summarizes fructose processing, glycerol-backbone supply, fatty acid availability, and TAG assembly. Human challenge studies support KHK dependence of PME and Pi responses; comparable dependence across the full ATP time course remains unresolved. Solid arrows indicate substrate flow or summarized biological relationships, without implying that each connection has been established in human MASLD. Orange dashed arrows indicate proposed links from gut-derived signaling inputs or hepatocyte injury signals to chronic inflammatory–fibrotic remodeling. The lower module summarizes interacting immune and stromal processes rather than a fixed cellular sequence. T-shaped ends indicate inhibition: PF-06835919 inhibits KHK activity, while the KHK-C–CPT1A/FAO connection summarizes an inhibitory effect reported in experimental models. Neither the dashed links nor the overall diagram establishes a continuous causal pathway from fructose intake to human fibrosis.

The first category comprises substrate-related inputs. Intact fructose entering the portal circulation directly supplies substrate for hepatic KHK. Its delivery, together with KHK expression, enzymatic activity, and downstream metabolic capacity, shapes the KHK–F1P response and the PME and Pi time courses. Experimental tracer studies further suggest that the proximal small intestine can partition part of fructose carbon into glucose, lactate, glycerate, and other organic acids. These metabolites may contribute to downstream hepatic carbon flux, but their quantitative contributions to human G3P formation, DNL, FFA re-esterification, and TAG assembly have not been established. Intrahepatic TAG accumulation therefore cannot be explained by DNL alone; glycerol-backbone supply, peripheral FFA flux, fatty acid oxidation, and very-low-density lipoprotein triacylglycerol (VLDL-TAG) export must also be considered.

The second category comprises gut-derived signaling inputs. Fructose that exceeds proximal intestinal processing capacity may reach the distal lumen and alter microbial substrate use, barrier status, and the delivery of microbe-derived molecules. These signals primarily shape the inflammatory context surrounding hepatocytes and immune cells and are not equivalent to the hepatic KHK substrate load. Substrate-related and gut-derived signaling inputs are distinguished here by their proposed roles and measurement compartments, rather than by demonstrated causal independence in humans. They may interact and converge at the level of hepatocellular injury and within the local immune environment. Their covariance, temporal order, and relative contributions remain unresolved. Simultaneous measurement could characterize their relationships, but establishing independent causal effects would additionally require interventions or other designs capable of separating the pathways.

The acute timescale spans minutes to hours and captures PME, Pi, and ATP responses after a fructose challenge, together with changes in plasma uric acid, lactate, glycerate, and FFAs. Stable-isotope tracing can further quantify DNL, formation of the TAG glycerol backbone, and FFA re-esterification flux. The chronic timescale spans weeks to years and includes adipose-tissue insulin resistance, sustained FFA release, organelle adaptation, microbiome and barrier remodeling, and the formation and stabilization of the inflammatory–fibrotic niche in MASH. Acute phosphorus metabolite responses reflect immediate substrate processing and pharmacodynamic effects; they cannot be equated with long-term histological outcomes.

The relationship between disease stage and fructose response should therefore be regarded as a prediction of the framework rather than an established causal relationship. Orange dashed connections in Figure 1 highlight the proposed links from gut-derived signaling inputs or hepatocyte injury signals to chronic inflammatory–fibrotic remodeling.

## 9 Discussion and translational perspectives

### 9.1 Implications for interpreting heterogeneous fructose responses

The framework suggests that comparable dietary fructose intake need not produce comparable hepatic substrate exposure or downstream injury. A useful comparison therefore asks where responses diverge: intestinal processing, hepatic carbon flux, peripheral FFA supply, or the inflammatory context. This distinction may help explain inconsistent findings across studies and guide the selection of measurements, but it does not yet define a clinically validated fructose-response phenotype.

Across the evidence base, human pharmacological challenge studies provide the clearest support for KHK-dependent PME and Pi responses, but not for their prognostic value. Experimental studies broaden the mechanistic possibilities through intestinal substrate partitioning, G3P–FFA coupling, and KHK-related organelle responses, although their relevance depends on the model and intervention. Important differences remain: KHK protein reduction and catalytic inhibition can produce divergent metabolic effects, while short-term human microbiome findings do not consistently reproduce the changes reported in rodents. These differences limit broad generalization and identify the conditions that future studies must compare. The main unresolved issue is whether the proposed metabolic and gut-derived relationships explain clinically meaningful differences in human disease outcomes.

### 9.2 Candidate fructose-response profiles and requirements for validation

Assessment of fructose responses in research settings should not rely on a single measure. It should cover four levels: exposure, proximal processing, dynamic metabolic response, and disease outcome. Exposure measures include added-sugar intake, sugar-sweetened beverages, the chemical form of the sugar, energy context, food matrix, and ingestion rate. Proximal-processing measures include intact fructose delivery and the circulating glycerate response proposed in experimental studies. Dynamic measures include the hepatic PME, Pi, and ATP time courses, together with DNL, TAG glycerol-backbone formation, FFA re-esterification, and VLDL-TAG export. Disease outcomes include magnetic resonance imaging–proton density fat fraction (MRI-PDFF), MASH activity, noninvasive fibrosis measures, histological changes, and clinical outcomes.

These measures reflect different compartments and time windows and are not interchangeable. Their interpretation should follow the distinctions defined in Section 7 and the technical constraints described in Section 2.4.

Candidate response profiles should meet at least four conditions before they are used for clinical stratification. First, they should be reproducible under standardized challenge conditions. Second, their responses to dose, exposure form, and mixed meals should be defined. Third, they should show longitudinal associations with liver fat, histological activity, or fibrosis outcomes in independent populations. Fourth, they should predict benefit from intervention beyond conventional metabolic risk factors. Until these requirements are met, “fructose-sensitive MASLD” should not be used as a clinical diagnostic category. The relevant measures are better considered a panel of research response features. The proposed measurement domains, corresponding methods, biological compartments, timescales, interpretive roles, and validation requirements are summarized in Table 2.

**Table 2:** Measurement domains for fructose-response assessment in MASLD research.

Measurement domain	Variables or markers	Methods	Compartment and timeframe	Interpretation	Validation needs
Exposure context	Added-sugar intake, sugar-sweetened beverages, fructose dose, chemical form, energy context, food matrix, ingestion rate, and exposure duration	Dietary records, food-frequency questionnaires, controlled feeding, or standardized challenge protocols	Dietary level; single exposure to years	Defines the exposure condition to which a study conclusion applies and separates isocaloric substitution from excess-energy effects.	Self-reported intake is error-prone; dietary intake does not directly measure portal or hepatic fructose exposure; fructose, sucrose, HFCS, juice, and whole fruit should not be treated as interchangeable.
Proximal intestinal processing	Intact fructose reaching the circulation; glucose, lactate, glycerate, and other organic acids; circulating glycerate response	Standardized fructose challenge, metabolomics, stable-isotope tracing, and, where feasible, portal-input estimation	Small intestine, portal circulation, and peripheral circulation; minutes to hours	Intended to assess proximal intestinal partitioning of fructose carbon. Circulating glycerate is an experimental readout whose specificity for intestinal processing has not been established in human MASLD.	Detailed carbon-flow evidence is mainly experimental; direct portal measurement in humans is difficult; circulating glycerate requires human reference ranges, reproducibility testing, and outcome prediction.
Hepatic KHK and phosphorus-metabolite dynamics	PME, Pi, and ATP amplitude, time to peak, area under the curve, recovery rate, and signal coupling	Standardized fructose challenge combined with hepatic ^31^P-MRS; pharmacodynamic KHK-inhibition studies	Liver; minutes to hours	PME and Pi provide the clearest human pharmacodynamic readouts of KHK-related fructose phosphorylation; ATP reflects a broader energetic response.	PME is a composite resonance and is not metabolite-specific F1P quantification; comparable KHK-specific pharmacodynamic evidence across the full ATP time course remains unestablished; diet, fasting, dose, voxel placement, sampling interval, and quantification strategy should be standardized.
TAG assembly and lipid flux	DNL, fructose-derived TAG glycerol backbone, peripheral FFA flux, FFA re-esterification, hepatic fat content, and VLDL-TAG export	Multi-tracer stable-isotope studies, plasma lipid kinetics, MRI-PDFF, and VLDL-TAG turnover assessment	Liver and peripheral circulation; hours to days	Separates newly synthesized fatty acids, glycerol-backbone supply, peripheral fatty-acid input, and hepatic export as contributors to net TAG accumulation.	The G3P-FFA coupling model is supported mainly by extreme mouse experiments; human studies should quantify multiple fluxes in the same protocol and avoid equating DNL with total liver-fat accumulation.
Gut-derived signaling input	Distal fructose exposure, microbial functional capacity, barrier status, microbe-derived molecules, and innate-immune response thresholds	Fecal metagenomics/metabolomics, permeability or barrier markers, microbe-derived molecule assays, peripheral immune-cell stimulation, and portal-related measures where feasible	Gut lumen, barrier, portal/peripheral circulation, and immune cells; hours to weeks	Captures how gut-derived signals modify the inflammatory context around hepatocytes and immune cells rather than directly measuring hepatic KHK substrate load.	Microbiome effects are strongly diet- and host-context dependent; human intrahepatic causal evidence is limited; peripheral immune-cell assays cannot substitute for MASH liver macrophage biology.
Disease activity and outcomes	MRI-PDFF, ALT, MASH activity, noninvasive fibrosis measures, histology, fibrosis progression, and clinical outcomes	MRI, laboratory tests, elastography, liver biopsy, and longitudinal follow-up	Liver and systemic disease level; months to years	Determines whether acute metabolic responses carry long-term disease meaning and whether interventions alter tissue-level outcomes.	Acute PME, Pi, or ATP changes should not be used as histological or fibrosis surrogate endpoints without validation; analyses should be stratified by disease stage and prespecified outcomes.
Clinical translation threshold	Repeatability, dose-response behavior, mixed-meal stability, independent prognostic value, and prediction of intervention benefit	Repeated challenges, independent cohorts, longitudinal modeling, and stratified intervention trials	Across compartments and timescales	Determines whether fructose-response features can move from mechanistic research tools to precision-nutrition or metabolic-targeting stratifiers.	Current evidence does not support “fructose-sensitive MASLD’ as a clinical diagnostic category; incremental value beyond conventional metabolic risk factors and liver disease stage must be shown.

PME, phosphomonoester; Pi, inorganic phosphate; ATP, adenosine triphosphate; DNL, *de novo* lipogenesis; FFA, free fatty acid; HFCS, high-fructose corn syrup; VLDL-TAG, very-low-density lipoprotein triacylglycerol; MRI-PDFF, magnetic resonance imaging-proton density fat fraction; ALT, alanine aminotransferase; MASLD, metabolic dysfunction-associated steatotic liver disease; MASH, metabolic dysfunction-associated steatohepatitis; KHK, ketohexokinase; ^31^P-MRS, phosphorus-31 magnetic resonance spectroscopy; F1P, fructose-1-phosphate; G3P, glycerol-3-phosphate; TAG, triacylglycerol; MRI, magnetic resonance imaging.

### 9.3 Intervention priorities: reducing substrate load, testing KHK inhibition, and strengthening intestinal buffering

Human dietary studies distinguish effects on metabolic flux from changes in hepatic lipid content. Controlled-feeding evidence indicates that energy balance and food source influence the hepatic effects of fructose-containing sugars; findings from excess-energy supplementation should therefore be distinguished from those of isocaloric substitution (23, 24). The beverage trial discussed in Section 3.1 demonstrated increased hepatic DNL after fructose or sucrose consumption in healthy men (36). This establishes a change in lipid synthesis under the tested conditions, but does not by itself demonstrate progressive steatosis or MASH. DNL measures one component of hepatic lipid metabolism, whereas liver-fat content reflects the net balance of fatty acid supply, synthesis, oxidation, and export.

The FRUITLESS trial provides complementary intervention evidence. Six weeks of fructose restriction produced a small additional reduction in intrahepatic lipid relative to an isocaloric comparator in adults with overweight and a high fatty liver index, without detectable improvements in glucose tolerance or serum lipid concentrations (81). These findings suggest that an effect on liver fat need not be accompanied by improvement across metabolic endpoints, and they do not establish histological MASH resolution or fibrosis regression. Reducing added sugars and sugar-sweetened beverages remains consistent with guideline-based care, particularly when their consumption contributes to excess energy intake (2, 3). Its expected benefit should be interpreted alongside total energy intake, weight change, food source, and baseline metabolic status.

Human intervention studies provide evidence that KHK inhibition can modify hepatic fat accumulation, although their populations and endpoints differ. Kazierad et al. randomized 53 adults with NAFLD in a placebo-controlled phase 2 trial; PF-06835919 at 300 mg reduced whole-liver fat relative to placebo, whereas the 75-mg dose did not produce a significant reduction (32). In a subsequent 16-week trial involving 164 participants with NAFLD and type 2 diabetes, Saxena et al. reported a significant reduction in MRI-PDFF-derived liver fat with 300 mg PF-06835919 versus placebo, while changes in HbA1c were not statistically significant (33). These findings distinguish an effect on hepatic fat from a consistent improvement across metabolic outcomes.

More recently, Koene et al. studied six weeks of PF-06835919 treatment in a randomized placebo-controlled crossover trial involving 15 participants with MASLD. The primary outcome was hepatic insulin sensitivity, assessed by insulin-stimulated suppression of endogenous glucose production. Treatment improved hepatic insulin sensitivity and reduced intrahepatic lipid without changing body weight; improvements in peripheral and adipose tissue insulin sensitivity and nocturnal fat oxidation were also reported (82).

Together, these trials extend the human evidence beyond acute pharmacodynamic responses to hepatic fat and insulin sensitivity. Their different populations, designs, durations, and endpoints limit direct comparison, and the small crossover trial requires confirmation in larger studies. These metabolic findings should remain distinct from evidence of histological MASH improvement or fibrosis regression. Earlier drug-development studies provide complementary evidence on pharmacokinetics, safety, and target engagement in experimental models and healthy participants (83).

Additional KHK inhibitors have entered translational development. LY3522348 is an oral dual inhibitor of KHK-A and KHK-C. In a first-in-human phase 1 study involving 65 healthy participants, single and repeated dosing was generally well tolerated, and a dose-dependent increase in plasma fructose after a fructose beverage supported inhibition of fructose metabolism (84). GS-1291269 is a potent, neutral KHK inhibitor with favorable preclinical pharmacokinetic properties; it reduced hepatic and renal F1P in a rat fructose-challenge model (85). These studies extend the evidence for pharmacological inhibition of fructose metabolism but do not establish efficacy against MASH or fibrosis: the LY3522348 trial enrolled healthy participants, whereas the cited GS-1291269 study provided preclinical evidence.

The Park comparison also cautions that reduced steatosis alone may obscure divergent metabolic effects. KHK-targeted strategies require separate assessment of protein reduction, catalytic inhibition, tissue distribution, and off-target activity (58). Isoform selectivity also warrants consideration, given the opposing effects of KHK-A and KHK-C reported in fructose-fed mice (30). The reported proteostatic role of KHK also supports assessing ER adaptation and hepatocyte viability alongside metabolic benefit, particularly when interventions reduce protein abundance (56). Evidence for histological improvement in MASH and definitive fibrosis endpoints remains limited. Long-term safety, tissue selectivity, renal or intestinal compensation, and the populations most likely to benefit remain important priorities.

Human studies show that microbiome-targeted diets can alter microbial composition and immune status, providing mechanistic support for dietary modulation of the gut-derived inflammatory context (86). Dietary fiber, microbial function, and barrier integrity therefore represent potential intervention targets, but their effects on fibrosis in human MASH require direct testing. These strategies currently address distal fructose spillover and the inflammatory context; no clinical evidence yet demonstrates fibrosis reversal in human MASH. The framework predicts that early substrate restriction or KHK targeting may more readily influence liver fat and metabolic responses. Whether established fibrosis requires combined immune–matrix intervention should be examined in trials stratified by disease stage.

## 10 Limitations and priority research questions

### 10.1 Limitations of the current evidence

The available evidence is uneven across study populations and endpoints. Human studies concentrate on acute fructose challenges, liver fat, and metabolic surrogate measures, whereas evidence for G3P–FFA coupling, circulating glycerate, and microbial buffering comes mainly from mice. Broader single-cell and spatial studies of the MASH niche provide a downstream disease context but do not directly measure fructose exposure.

Animal models differ substantially in dietary composition, fructose proportion, exposure duration, and genetic background (43). Pure fructose, sucrose, high-fructose corn syrup, GAN diets, and high-fat, high-fructose diets create distinct metabolic environments. High-dose liquid-fructose challenges are useful for amplifying pharmacodynamic signals, but their generalizability is largely limited to comparable experimental conditions.

Studies use different endpoints, including PME, Pi, ATP, liver fat, ALT, inflammatory molecules, histology, and fibrosis, each representing a different level of evidence. The technical constraints described in Section 2.4 further limit cross-study comparability. No human cohort has yet combined a standardized fructose challenge, ^31^P-MRS, stable-isotope tracing of fructose carbon flux and FFA flux, intestinal barrier assessment, intrahepatic immune phenotyping, and longitudinal fibrosis follow-up. Prospective studies are needed to test the validity of links across these levels.

This narrative review does not provide pooled effect estimates, a formal risk-of-bias assessment, or GRADE certainty ratings. Its interpretations therefore rely on qualitative comparisons of study design, experimental context, consistency, and applicability to human MASLD. Limitations in literature availability, possible publication bias, and heterogeneity among studies may affect these interpretations; the framework should not be regarded as a formally graded body of evidence.

### 10.2 Knowledge gaps and priority research questions

The framework requires tests of its individual links before it can support clinical stratification. Table 2 summarizes the measurement domains; the priorities below specify study designs and observations that could support or challenge the proposed relationships.

In a mouse study, the circulating glycerate response to oral fructose was associated with intestinal fructose catabolism and resistance to fructose-induced hepatic steatosis (22). Intestinal partitioning should first be tested under controlled exposure conditions. Randomized crossover studies using labeled fructose could compare dose, ingestion rate, and food matrix while measuring circulating fructose-derived metabolites and hepatic metabolic responses. Experimental studies with intestine-specific KHK manipulation could distinguish reduced hepatic fructose delivery from other intestinal effects. A consistent relationship between circulating glycerate and independently estimated intestinal fructose catabolism would support its proposed marker role. Poor reproducibility or dissociation from intestinal processing would limit that interpretation.

The meaning of hepatic phosphorus metabolite responses requires repeated standardized challenges combined with ^31^P-MRS and longitudinal follow-up. PME, Pi, and ATP amplitude, timing, and recovery should be evaluated separately against liver fat, disease activity, and fibrosis outcomes, accounting for exposure conditions and baseline metabolic status. In participants with MASLD, PF-06835919 suppressed the fructose-induced hepatic PME peak and transient Pi decline, supporting their use as pharmacodynamic readouts of KHK inhibition. This finding does not establish their prognostic value (26). If these responses add no reproducible information beyond conventional risk factors and disease stage, their use for clinical stratification would remain unsupported.

In mice with liver-specific *Irs1/Irs2* deletion and high-fructose exposure, isotope tracing implicated fructose-derived glycerol-backbone supply in hepatic TAG assembly under conditions of increased FFA availability (42). Human multi-tracer studies are needed to test G3P–FFA coupling by quantifying fructose-derived TAG glycerol-backbone formation, DNL, peripheral FFA flux, and VLDL-TAG kinetics within the same protocol. The relevant question is whether glycerol-backbone supply and FFA availability jointly contribute to TAG assembly under defined metabolic conditions. Little fructose-derived glycerol incorporation, or no detectable coupling with FFA availability despite adequate measurement sensitivity, would argue against a substantial role for this mechanism in the population studied.

Substrate-related and gut-derived signaling inputs should also be measured together. Controlled dietary studies could combine metabolic tracing with microbial functional profiling, barrier-related measures, and immune-response assays. Selective perturbation of these pathways in experimental models would help assess whether they make distinguishable contributions to hepatic injury. Peripheral inflammatory changes alone would not establish intrahepatic effects, and tightly correlated inputs without selective perturbation would leave their independent contributions unresolved.

Finally, the proposed connection to inflammatory-fibrotic remodeling requires disease-stage-specific testing. Experimental models should combine defined fructose exposure with metabolic flux measurements, hepatic immune and HSC phenotyping, and histological assessment. Human longitudinal or intervention studies could relate exposure and response measures to disease outcomes, using liver tissue only when clinically and ethically appropriate. Improvement in acute metabolic responses without corresponding changes in inflammatory or fibrotic outcomes would weaken the proposed link in that setting and caution against using acute readouts as disease surrogates.

## 11 Conclusion

The proposed dual-input, dual-timescale framework organizes heterogeneous fructose responses in MASLD around intestinal processing, hepatic substrate use, and the inflammatory context. It identifies links for testing rather than an established causal sequence from fructose exposure to fibrosis.

Human challenge studies support KHK-dependent PME and Pi responses as pharmacodynamic readouts. The clinical value of broader response profiles remains unresolved. Progress requires determining whether these profiles provide reproducible information about disease outcomes and intervention benefit beyond conventional metabolic risk factors.
